# Bridging the gap: Essential factors for poststroke community reintegration in low-and-middle income countries

**DOI:** 10.1371/journal.pone.0336517

**Published:** 2025-12-10

**Authors:** Fareeha Noor Farooq, Akshatha Nayak, Vijay Kumar K, Srikant Natarajan, Shivanand Pai, Rinita Mascarenhas

**Affiliations:** 1 Department of Physiotherapy, Kasturba Medical College Mangalore, Manipal Academy of Higher Education, Manipal, India,; 2 Department of Oral Pathology and Microbiology, Manipal College of Dental Sciences Mangalore, Manipal Academy of Higher Education, Manipal, India; 3 Department of Neurology, Kasturba Medical College Mangalore, Manipal Academy of Higher Education, Manipal, India; 4 Department of Neurology, Christian Medical College & Hospital, Ludhiana, India; University of Birmingham, UNITED KINGDOM OF GREAT BRITAIN AND NORTHERN IRELAND

## Abstract

**Background:**

Community reintegration (CR), a fundamental goal in stroke rehabilitation, depends on both patients’ personal factors and disease-related factors. Dissatisfaction in reintegration levels is commonly observed among chronic stroke patients; hence, it is essential to understand the factors influencing CR of the chronic stroke patients in low-and-middle income countries.

**Methods:**

A cross-sectional study was carried out involving 53 chronic stroke patients residing in the community aged over 18 years, with a single episode of stroke. Demographic details and patient-reported outcomes, including community reintegration, perceived social participation, balance self-efficacy, fear of fall, physical function, quality of life, caregiver strain, and perceived social support, were collected. The outcomes were correlated using Karl Pearson’s correlation for continuous and the t-test for categorical variables. Furthermore, a multiple linear regression was employed to identify the strongest predictor.

**Results:**

All the patient-reported outcomes assessed in the present study have a significant influence on CR. A multiple regression analysis revealed that perceived social participation (β = −4.039, *p* < 0.001), physical function (β = 0.470, *p* = 0.018), and caregiver strain (β = −0.241, *p* = 0.040) were good predictors of CR. However, perceived social participation restriction was observed to be the strongest predictor (R^2^ = 0.812).

**Conclusion:**

This study identified balance self-efficacy, physical function, quality of life, and perceived social support as positive contributors to CR, while fear of fall, caregiver strain and reduced perceived social participation hindered CR. Findings highlight the need for culturally sensitive strategies, especially in lowmiddle income countries, where family-led caregiving is predominant. Community-based programs and inclusive digital platforms may enhance recovery while empowering both patients and caregivers.

## Introduction

The WHO’s *Rehabilitation 2030: A call for action* emphasizes the urgent need to strengthen rehabilitation services that are person-centered, integrated into health systems, and responsive to long-term functional needs, particularly in low-and-middle income countries. In alignment with this global vision, one of the objectives of stroke rehabilitation is often community reintegration (CR) [1]. CR can be described as the ability of an individual to engage more actively in both leisure and productive activities as part of their expected role in the community [2]. Many subjects reported low levels of satisfaction with their CR, which aligns with their self-perceived participation in the community [3].

The impact of stroke is unexpected, forcing sudden changes in the overall psychology and behavior of stroke survivors [4]. It is important to acknowledge the complexities related to physical, verbal and social barriers that may restrict people with disabilities from being able to participate within their community [5]. The complex interplay between functional, personal, and environmental factors creates significant challenges in daily activity participation and self-efficacy. Compared with individuals without a stroke, they often experience unsatisfactory levels of community participation [6]. This dissatisfaction is intricately linked with variables such as age, duration of stroke, balance self-efficacy and ability, anxiety, depression, quality of life (QOL), return to work, and walking ability [7]. Balance self-efficacy refers to the confidence an individual has in performing tasks without losing balance and has been shown to be compromised among chronic stroke patients [8]. Restrictions in functional mobility are a significant barrier that hinders participation in activities and further limits reintegration into the community [9].

In terms of psychosocial factors, both anxiety and depression are predominantly seen where anxiety is identified as a response to apprehension, whereas depression is the result of potential disability and limitations in lifestyle [10]. These factors have been linked to slow recovery, poor functional outcomes, and overall lower QOL [11]. Support from informal caregivers is a crucial factor in reintegrating the individual into the community and maintaining the initial gains from rehabilitation [4]. The priorities of patients differ from those of healthcare teams. While healthcare teams focus primarily on the execution of physical tasks and functions, patients focus on the social context and return to normality. Therefore, to achieve the most satisfying results of rehabilitation, it is necessary to consider patient’s perspective on CR and the social context, respectively [12].

Low-and-middle income countries face a unique set of challenges in facilitating CR for stroke survivors [13]. In India, stroke survivors with poor functional independence tend to exhibit lower levels of CR [14]. Among the factors studied in subacute stroke patients, it was found that physical function, balance self-efficacy, and fear of falling significantly influence CR [15]. Approximately 60–85% of stroke survivors regain the ability to walk independently within six months after stroke, and walking is a fundamental step towards achieving functional independence [16]. Invariably, studies have reported that better functional independence is associated with improved CR [14,15].

Although various physical, psychosocial, and contextual factors have been studied individually in chronic poststroke populations, a comprehensive approach to understanding their combined influence remains inadequate. Thus, the underexplored and unique social dynamics of low-and-middle income countries warrant a deeper understanding to enhance healthcare delivery and advance overall public health outcomes.

## Materials and methods

This study was approved by the Ethics Committee of Kasturba Medical College Mangalore, India (IEC KMC MLR 12/2023/488) on December 21, 2023. All participants provided written informed consent in a language they understood prior to enrollment in the study, and the study was conducted ethically in accordance with the World Medical Association Declaration of Helsinki. The study was conducted from January 1, 2024 to March 31, 2025.

Based on a previous study [15], the correlation coefficient between the key variables was reported to be –0.61, corresponding to a coefficient of determination (R²) of 0.3721. Using this effect size, with an alpha error of 1% (two-tailed) and a power of 99.5%, the base sample size was estimated using the formula:


$$N=((Z_{\text{1}}-\frac{\alpha }{\text{2}}+Z_{\text{1}}-\beta ){)}^{\text{2}}\times \;(\text{1}\;-\;\text{R}²))\;/\;\text{R}²$$

Substituting Z₁ ₋ ᾳ/₂ = 2.5758 and Z₁ ₋ β = 2.5758, the calculated base sample size (N₀) was 44.78. To account for multiple predictors in a regression model, the formula N = N₀ + (m + 1) was applied, where m represents the number of predictors. Considering up to six predictor variables, the adjusted required sample size was approximately 51.78, which was rounded up to 52. Therefore, the final sample size of 53 participants used in the study was adequate to detect the previously reported large effect size (r = –0.61) with the specified alpha level and statistical power. This sample size is thus sufficient to support a multiple regression model including up to six predictors.

**Fig 1:**
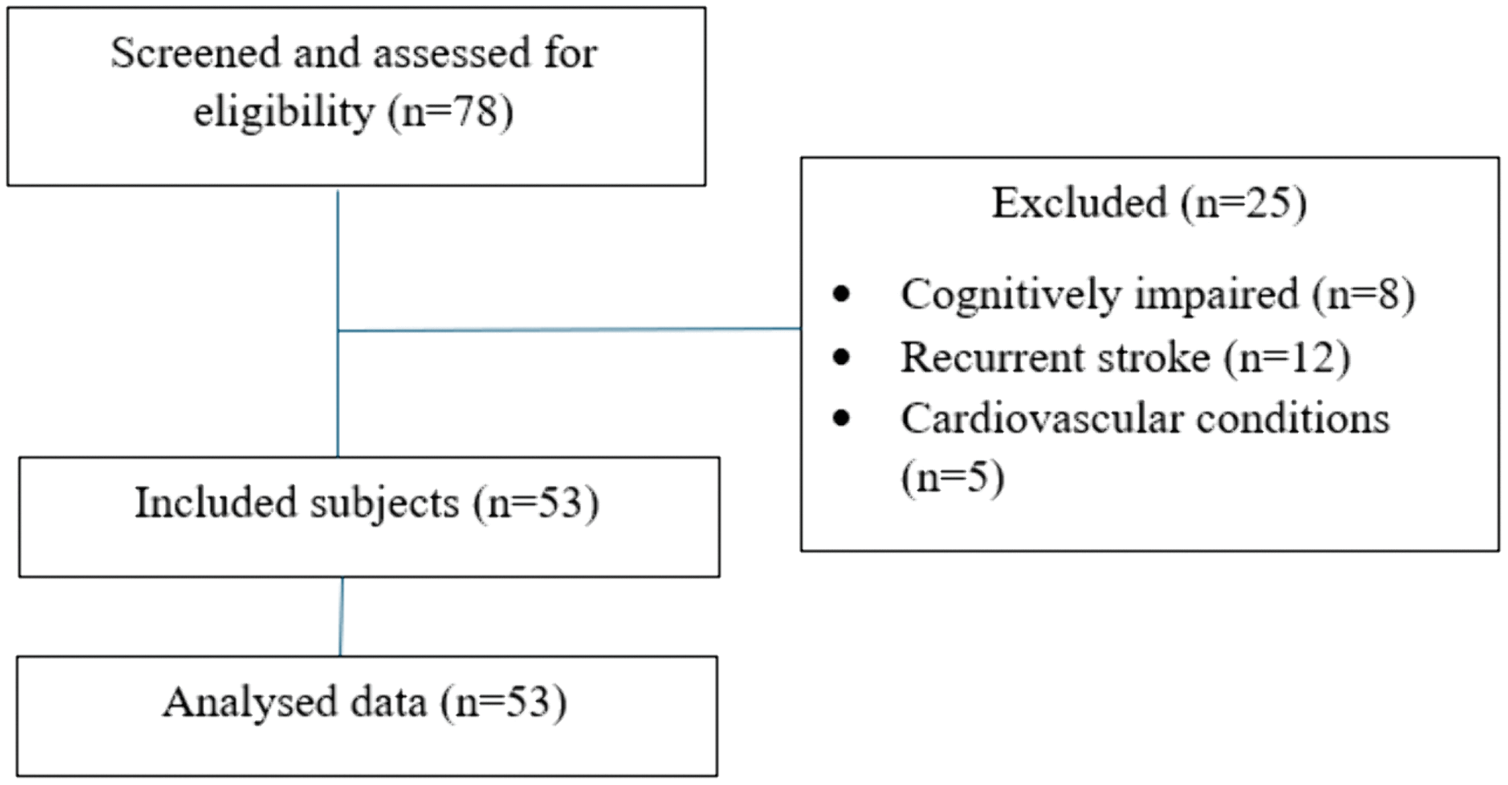
Participant flowchart.

A community-based cross-sectional study was conducted using a tertiary hospital database to identify community-dwelling poststroke patients who were initially admitted to the hospital for care and had been residing in their homes for more than 6 months poststroke, as confirmed via telephonic contact. The participants were selected by convenience sampling and were further approached and explained the study, and written informed consent was obtained. The inclusion criteria were as follows: (1) individuals who were diagnosed with a single episode of stroke by a neurologist, (2) adult stroke patients aged above 18 years, and (3) individuals with a poststroke duration > 6 months. Participants were excluded if their cognition was impaired (score < 26 on the Montreal Cognitive Assessment) or if they had musculoskeletal conditions (such as osteoarthritis) or cardiovascular conditions that may have interfered with the study parameters (Fig 1).

The details of the participants, namely, their age, sex, type of stroke, side affected, poststroke duration, and presence of comorbidities (diabetes mellitus/hypertension), were collected. The outcome variables included physical outcomes such as balance self-efficacy, fall efficacy, physical function, psychosocial outcomes such as stroke-specific QOL, perceived social support, caregiver strain, perceived social participation, and CR.

All outcome measures used in this study are free to use. The tools were administered by the interviewer over a duration of 25–30 minutes.

### Assessment

#### Community reintegration.

CR was assessed via the Reintegration to Normal Living Index (RNLI), which consists of eleven items formed by statements reflecting the patient’s situation. Each item is scored on a 10-point visual analog scale, where 0 represents minimal reintegration and 10 represents complete reintegration. This scale has been shown to have good test‒retest reliability (ICC: 0.83‒0.87) and good internal consistency (Cronbach’s α: 0.73‒0.97) [17,18].

#### Perceived social participation.

The Participation Scale (P-Scale), comprising 13 items, was used to assess individuals’ perceptions of social integration and participation within their communities. Each question measures the level of participation in comparison to peers. The scale showed to be psychometrically sound, with an internal consistency of 0.92, an intra-tester stability of 0.83 and an inter-tester reliability of 0.80 [19].

#### Balance self-efficacy.

The Activities-specific Balance Confidence (ABC) scale is a questionnaire that individuals use to rate their confidence in performing specific activities without losing balance. A lower score indicates lower confidence, whereas a score of 100 indicates full confidence. The ABC is an acceptable outcome measure, with internal consistency values of 0.94 and 0.85 for test-retest reliability [20,21].

#### Fear of Fall.

Fall Efficacy Scale–International (FES-I) is a questionnaire used to assess fear of falling, scored according to the individual’s level of concern about falling while performing a specific activity. The higher the score is, the greater the fear of falling. The FES-I was found to have a good overall structure, with excellent test‒retest reliability of 0.94 and 0.96, indicating high internal consistency [22,23].

#### Physical function.

The Stroke Impact Scale-16 (SIS-16) comprises 16 items in the physical domains of strength, hand function, mobility, and activities of daily living, derived from the Stroke Impact Scale (SIS). It stands as a brief tool for measuring stroke recovery in terms of physical ability. The scale had a Cronbach’s alpha of 0.94 and an intraclass correlation coefficient of 0.95, thus supporting its use in both clinical and research settings [24–26].

#### Quality of life.

The Stroke-Specific Quality of Life (SS-QOL) has been established as a valid and reliable measure of stroke-specific health-related QOL. The items fall under the following domains: mobility, energy, upper extremity function, work and productivity, mood, self-care, social roles, family roles, vision, language, thinking, and personality. The higher the score is, the better the functioning. With respect to psychometric properties, the scale demonstrated Cronbach’s alpha values ranging from 0.65 to 0.94 and intraclass correlation coefficients from 0.70 to 0.91, indicating acceptable reliability for use [26,27].

#### Caregiver strain.

The Modified Caregiver Strain Index (MCSI) is a tool for screening caregiver strain in a long-term context. The following domains are included: financial, physical, psychological, social, and personal. The level of caregiver strain is indicated by higher scores. The MCSI has slightly better internal reliability (α = .90) than the original Index, with a two-week test–retest reliability of 0.88 [28].

#### Perceived social support.

The Multidimensional Scale of Perception Social Support (MSPSS) is a tool used to assess the individual’s perception of overall social support. It measures the support received from friends, family, and the individual’s significant other. The scale has been reported to have internal reliability ranging from 0.84 to 0.92, and both the factor structure and validity of the scale have been confirmed [29].

### Statistical analysis

The data were entered and analyzed using the Statistical Package for Social Sciences (IBM SPSS Statistics for Windows Version 29.0, Armonk, NY). Categorical variables are reported as frequencies, whereas continuous variables are summarized in terms of means and standard deviations. Furthermore, Karl Pearson’s correlation was used to establish the correlation of continuous variables with CR, and an independent t test was used for categorical variables. Multiple linear regression was used to identify the predictors of CR, with *p* < 0.05 considered significant.

## Results

The study included 53 poststroke patients, 43 of whom were males (mean age 57.3 years) and 10 of whom were females (mean age 54.5 years), with an overall mean poststroke duration of 26.6 months. With respect to the type of stroke, 34 had ischemic strokes, whereas 19 experienced hemorrhagic strokes. Regarding the affected side, 28 patients experienced right hemiparesis, and 25 had left hemiparesis. Additionally, 31 patients were diabetic, and 31 were hypertensive (Table 1).

**Table 1:** Descriptive statistics of study participants.

Variable		N (%)
**Gender**	Male	43 (81.13%)
	Female	10 (18.87%)
**Type of stroke**	Ischemic	34 (64.15%)
	Hemorrhagic	19 (38.85%)
**Side affected**	Right	28 (52.83%)
	Left	25 (47.16%)
**Diabetes mellitus (DM)**	Present	31 (58.49%)
	Absent	22 (41.5%)
**Hypertension (HTN)**	Present	31 (58.49%)
	Absent	22 (41.5%)
	**Mean±SD**	
**Age** (in years)	56.8 ± 10.2	
**Poststroke duration** (in months)	26.6 ± 18.1	
**RNLI**	79.00 ± 20.70	
**P scale**	20.47 ± 11.62	
**ABC**	64.47 ± 20.62	
**FES-I**	31.41 ± 11.88	
**SIS-16**	63.92 ± 12.11	
**SS-QOL**	184.32 ± 37.08	
**MCSI**	5.83 ± 5.58	
**MSPSS**	66.96 ± 14.45	

SD, Standard Deviation; N, number of subjects; RNLI, Reintegration to Normal Living Index; P scale, Participation scale; ABC, Activities-specific Balance Confidence scale; FES-I, Fall Efficacy Scale – International; SIS-16, Stroke Impact Scale – 16; SS-QOL, Stroke Specific – Quality of Life; MCSI, Modified Caregiver Strain Index; MSPSS, Multidimensional Scale of Perceived Social Support.

All outcomes demonstrated significant correlations with CR, except for age and poststroke duration, which exhibited weak and nonsignificant correlations (*p* > 0.05). Balance self-efficacy (r = 0.732), physical function (r = 0.755), QOL (r = 0.742), and perceived social support (r = 0.462) were positively correlated with CR (*p* < 0.001). Additionally, perceived social participation (r = −0.746), FOF (r = −0.658) and caregiver strain (r = −0.708) were negatively correlated, i.e., increased perceptions of social restrictions, high FOF and high levels of caregiver strain were linked to poorer CR (Table 2).

**Table 2:** Correlation of outcome variables with CR.

Variable	RNLI	
	*r* value	*p* value
**Age** (in years)	0.059	0.675
**Poststroke duration** (in months)	0.109	0.435
**P scale**	−0.746	**<0.001**
**ABC**	0.732	**<0.001**
**FES-I**	−0.658	**<0.001**
**SIS-16**	0.755	**<0.001**
**SS-QOL**	0.742	**<0.001**
**MCSI**	−0.708	**<0.001**
**MSPSS**	0.462	**0.001**

RNLI, Reintegration to Normal Living Index; P scale, Participation scale; ABC, Activities-specific Balance Confidence scale; FES-I, Fall Efficacy Scale – International; SIS-16, Stroke Impact Scale – 16; SS-QOL, Stroke Specific – Quality of Life; MCSI, Modified Caregiver Strain Index; MSPSS, Multidimensional Scale of Perceived Social Support; *p* < 0.05 considered significant.

Variables such as sex, type of stroke, side affected, and the presence of diabetes or hypertension were not significantly associated with CR, indicating that these factors did not show independent associations with return to normal living.

A forward stepwise multiple linear regression was performed to identify the predictors of CR, where three variables were retained in the final model: physical function, perceived social participation, and caregiver strain.

The model demonstrated strong predictive ability (R = 0.884), with an R² of 0.782, indicating that 78.2% of the variance in CR levels was explained by these predictors. The adjusted R² was 0.768, accounting for model complexity, and the standard error of the estimate was 9.963, suggesting a good model fit.

Among the predictors, physical function showed a significant positive association with CR (B = 0.692, β = 0.408, t = 4.755, *p* < 0.001), indicating that higher stroke impact scores were associated with better reintegration. In contrast, both perceived social participation (B = –0.723, β = –0.410, t = –4.928, *p* < 0.001) and caregiver strain (B = –0.878, β = –0.237, t = –2.646, *p* = 0.011) demonstrated significant negative associations, suggesting that greater participation restrictions and higher caregiver strain were linked to poorer reintegration outcomes.

Collinearity diagnostics confirmed acceptable levels of multicollinearity, with tolerance values ranging from 0.556 to 0.645 and variance inflation factor (VIF) values between 1.551 and 1.799, all within acceptable limits (< 10).

**Fig 2:**
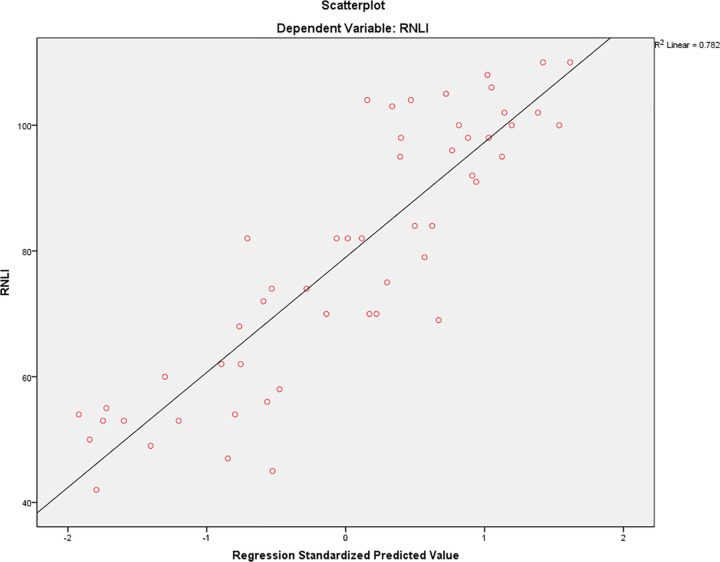
Regression scatterplot.

Overall, the forward stepwise regression model identified physical function, perceived social participation and caregiver strain as significant predictors of reintegration to normal living among poststroke individuals, collectively explaining 78.2% of the variance in CR scores while maintaining low collinearity among the variables. (Table 3) (Fig 2).

**Table 3:** Forward stepwise multiple linear regression.

Model		Unstandardized Coefficients		Standardized Coefficients	t	*p* value	95% Confidence Interval		Statistics	
		B	Standard Error	Beta			Lower Bound	Upper Bound	Tolerance	VIF
3	Constant	54.707	11.260		4.859	**<0.001**	32.080	77.334		
	SIS-16	0.692	0.145	0.408	4.755	**<0.001**	0.399	0.984	0.604	1.657
	P scale	−0.723	0.147	−0.410	−4.928	**<0.001**	−1.018	−0.428	0.645	1.551
	MCSI	−0.878	0.332	−0.237	−2.646	**0.011**	−1.545	−0.211	0.556	1.799
Dependent Variable: RNLI **RNLI = 54.707 + (0.692 × SIS-16) – (0.723 × P scale) – (0.878 × MCSI)** Model summary: R = 0.884, R² = 0.782, Adjusted R² = 0.768, Standard error of the estimate = 9.963.										

RNLI, Reintegration to Normal Living Index; SIS-16, Stroke Impact Scale – 16; P scale, Participation scale; MCSI, Modified Caregiver Strain Index; *p* < 0.05 considered significant.

## Discussion

This study aimed to investigate the relationships between CR and various factors, with a focus on identifying the strongest predictors of CR in patients with chronic stroke. The results revealed that factors such as perceived social participation, balance self-efficacy, FOF, physical function, QOL, caregiver strain, and perceived social support were significantly associated with CR. With better levels of balance self-efficacy, physical function, QOL, and perceived social support, CR increased in such individuals. However, more restrictions in perceived social participation, increased FOF, and greater burden on caregivers were linked with poorer CR. Perceived social participation and physical function emerged as the strongest predictors of reintegration, along with caregiver strain.

Previous studies have reported conflicting evidence regarding that relationship between age and CR [30], and age is also considered a good predictor in the early phase of recovery [31]. The individuals in the present study were in the chronic phase of stroke recovery, which may have been the possible reason for the poor relationship between age and CR. Depression is common among female stroke survivors and negatively impacts their recovery and CR [30]. In the present study, no difference was found in the CR between genders, which may be due to the underrepresentation of females in the study population. The type of stroke was not a significant predictor of outcome in this population, similar to previous studies that reported mixed evidence on the effect of stroke subtype on functional outcomes [14]. The presence of comorbidities has been acknowledged to adversely affect the level of reintegration among poststroke patients [32]. In the present study, however, diabetic and non-diabetic patients did not differ much in terms of their level of reintegration. The current literature shows that impaired glucose metabolism impacts neural function by disrupting neuroplasticity and hindering recovery after stroke. With increased HbA1c levels, poorer recovery poststroke is observed. However, this association remains inconclusive due to conflicting findings across studies [33]. Additionally, the results showed that individuals with hypertension were associated with poorer reintegration. Chronic hypertension has been associated with cognitive decline, mainly due to disruption of vascular reactivity and neurovascular coupling [34]. Additionally, elevated blood pressure has a chronic impact on both balance and gait over time in older adults [35].

The findings of this study indicated that perceived social participation, physical function, and the burden of caregivers had significant influences on CR. Disconnection from oneself poststroke is a major barrier to recovery, which limits one’s ability to engage in social interactions and reaching pre-event status [12]. This goal of recapturing self-identity directly links to the reintegration process, which involves rebuilding of autonomy and independence in the individual [36]. This relationship suggests that higher levels of perceived social restriction are associated with lowered CR. Community ambulation and the ability to involve in social activities (visiting local shops, attending family functions, social gatherings, religious festivities) were prioritized by individuals of this population, which reflected a sense of belonging in the community. The results underscore the need to address these participation barriers as part of the rehabilitation planning.

Studies have reported that balance self-efficacy is a significant predictor of CR in poststroke patients [30]. In cases where balance self-efficacy is reduced, it may lead to lower activity levels, which may not directly linked to physical impairments but rather to self-imposed limitations [3]. The present study revealed that patients who were more confident in maintaining balance while performing their daily activities were associated with better CR. Another psychological barrier contributing to self-efficacy is FOF [3]. This may lead to the avoidance of performing activities to prevent falls [9], such as ambulating on uneven surfaces and sloped roads, which are commonly encountered in developing countries. Many participants of this study did not have good access to cemented roads in their community, which remained a major barrier to their daily participation. This contributes to poor functional performance and limitations in daily activities, resulting in poor reintegration, which the results of the present study also support. Our study also revealed that participants with lower confidence and higher FOF were apprehensive in unfamiliar environments, which led to limitations in community ambulation and causing overall restrictions in their participation in the community. Thus, rehabilitation strategies focusing on improving balance confidence, transference and safe mobility across various environments may improve CR.

Individuals with better physical ability and functional performance have been shown to yield better outcomes in CR [14,37]. The findings of the present study also show that participants with higher levels of physical function had higher levels of CR. This can be attributed to good motor recovery in this population, which makes them more physically able and, therefore, more independent in their functional abilities [14].

A good proportion of individuals poststroke present with poor QOL, mainly due to the residual effects of the stroke and the functional restrictions they face in their daily task performance [4]. QOL has been established to be intricately linked to functional recovery and the ability to engage in meaningful daily activities [38]. CR, as a reflection of QOL, encompasses various domains, including physical, psychological, and notably socio-cultural. A key source of satisfaction in life lies in the individual’s ability to assert autonomy and maintain their personal identity. Participants primarily exhibited lower self-esteem and a negative self-image, which often led them to avoid the outdoors and community interactions, possibly stemming from cultural beliefs and feelings of being different from others.

Stroke survivors often depend on caregivers to manage their disabilities and achieve normalcy [4]. While caregiving for patients is largely undertaken by families, greater professional involvement, awareness and understanding of the disease and its consequences remains inadequate in low-and-middle income countries. This results in stigmatization, which may include societal shunning and neglect due to local cultural ideologies and misunderstandings about the disease [39]. Study findings indicate that greater caregiver burden was linked to poorer reintegration outcomes, where the family was unable to handle frustrations related to a change in personality or monetary burden as well. In contrast, positive reinforcement by the family and robust social support from friends and the community have been identified as powerful enablers of reintegration among poststroke patients [38,40]. This can only be achieved with good education, community awareness, and reducing the burden on caregivers. The findings of the present study reinforced this observation, with the agreement that patients with greater perceived social support were more likely to experience better reintegration outcomes. In the Indian socio-cultural context, caregiving is influenced by family values and filial piety, and is not universally perceived as a burden [39]; therefore, supporting caregivers of this population may have a more positive impact on overall outcomes. Further, stroke support groups can significantly mitigate caregiver burden by fostering peer connection, offering access to care professionals, promoting stress management strategies, and addressing emotional isolation that often arises from the significant responsibilities of caregiving [41]. While digital technologies have increasingly been implemented, establishing a culturally attuned and relevant online support systems for caregivers is essential to overcoming such barriers [39] and contributing to a nationwide dissemination of knowledge and public sensitization.

## Limitations of the study

Firstly, the relatively small sample size restricts the generalizability of the findings; thus, future studies may opt for a multicenter design to enhance external validity. Secondly, women were underrepresented in the sample, reflecting the gender disparity often encountered in stroke care, which may limit applicability among the genders. Third, details on environmental barriers and socioeconomic status were not thoroughly explored. Although stroke severity at discharge was initially considered, incomplete details for some participants led to omission from the proforma. Similarly, psychological well-being was evaluated as a quality of life assessment component, but specific measures for anxiety and depression were not considered. Finally, recruitment primarily from a hospital database may have introduced potential selection bias and is considered a limitation of this study.

## Conclusion

The present study highlighted various factors influencing CR in chronic stroke patients and identified potential facilitators for effective rehabilitation in this population. A greater CR was observed in patients with better balance self-efficacy, physical function, QOL, and perceived social support. Additionally, greater restrictions in perceived social participation, FOF, and caregiver burden contributed to lower CR.

These findings underscore the importance of poststroke rehabilitation programs in prioritizing the improvement of balance, self-efficacy, and physical function of patients, while addressing functional outcomes and strengthening support systems.

In context of low-and-middle income countries, where health infrastructure is limited and caregiving is primarily dependent on family and community support, tailoring strategies to local socio-cultural dynamics is essential. Future policy making must focus on the development of community-based programs that raise awareness and promote public sensitization towards stroke and its complications, establishing culturally sensitive support groups, and create inclusive digital platforms that empower both patients and caregivers. Such initiatives may have the potential to bridge the gaps in rehabilitation delivery, reduce caregiver strain, and support long term recovery in low-resource settings.

## Supporting information

S1 File Dataset. (XLSX)

## References

[1] World Health Organization. Rehabilitation 2030: a call for action [Internet]. Geneva: WHO; [cited 2025 May 15]. Available from: https://www.who.int/initiatives/rehabilitation-2030

[2] Holavanahalli RK, Badger K, Acton A. Community Reintegration. J Burn Care Res. 2017;38(3):e632–4. doi: 10.1097/BCR.0000000000000563 28368917

[3] Pang MYC, Eng JJ, Miller WC. Determinants of satisfaction with community reintegration in older adults with chronic stroke: role of balance self-efficacy. Phys Ther. 2007;87(3):282–91. doi: 10.2522/ptj.20060142 17284545

[4] Okoye EC, Okoro SC, Akosile CO, Onwuakagba IU, Ihegihu EY, Ihegihu CC. Informal caregivers’ well-being and care recipients’ quality of life and community reintegration - findings from a stroke survivor sample. Scand J Caring Sci. 2019;33(3):641–50. doi: 10.1111/scs.12657 30734330

[5] Currie S, Douglas J, Winkler D. “What’s next?” The journey from hospital to community engagement from the perspectives of adults following severe acquired brain injury: a scoping review protocol. BMJ Open. 2022;12(9):e064226. doi: 10.1136/bmjopen-2022-064226 36130757 PMC9494587

[6] Shrivastav SR, Ciol MA, Lee D. Perceived Community Participation and Associated Factors in People With Stroke. Arch Rehabil Res Clin Transl. 2022;4(3):100210. doi: 10.1016/j.arrct.2022.100210 36123973 PMC9482037

[7] Akosile C, Nworah C, Okoye E, Adegoke B, Umunnah J, Fabunmi A. Community reintegration and related factors in a Nigerian stroke sample. Afr Health Sci. 2016;16(3):772–80. doi: 10.4314/ahs.v16i3.18 27917211 PMC5111998

[8] Tang A, Tao A, Soh M, Tam C, Tan H, Thompson J, et al. The effect of interventions on balance self-efficacy in the stroke population: a systematic review and meta-analysis. Clin Rehabil. 2015;29(12):1168–77. doi: 10.1177/0269215515570380 25681409 PMC4596690

[9] Olawale OA, Usman JS, Oke KI, Osundiya OC. Evaluation of Predictive Factors Influencing Community Reintegration in Adult Patients with Stroke. J Neurosci Rural Pract. 2018;9(1):6–10. doi: 10.4103/jnrp.jnrp_386_17 29456337 PMC5812161

[10] Murtezani A, Hundozi H, Gashi S, Osmani T, Krasniqi V, Rama B. Factors associated with reintegration to normal living after stroke. Med Arh. 2009;63(4):216–9. 20088178

[11] Obembe A, Mapayi B, Johnson O, Agunbiade T, Emechete A. Community reintegration in stroke survivors: Relationship with motor function and depression. Hong Kong Physiotherapy Journal. 2013;31(2):69–74. doi: 10.1016/j.hkpj.2013.04.001

[12] Wood JP, Connelly DM, Maly MR. “Getting back to real living”: A qualitative study of the process of community reintegration after stroke. Clin Rehabil. 2010;24(11):1045–56. doi: 10.1177/0269215510375901 20713436

[13] Gandhi DBC, Mascarenhas R, Zarreen S, Chawla NS, Pandian JD, English C, et al. Bridging the gap: unique strategies to improve access and implementation of stroke rehabilitation in LMICs - a scoping review. Disabil Rehabil. 2025;:1–13. doi: 10.1080/09638288.2025.2495194 40336256

[14] Mohakud K, Das SP, Sahoo S, Sahu S. Functional Performance and Community Reintegration of Chronic Post-stroke Survivors in Eastern India. JMR. 2023. doi: 10.18502/jmr.v17i3.13069

[15] Nayak A, Misri Z, Choezom, Vasyani MS, Unnikrishnan B, Joshua AM, et al. Reviewing Factors That Predict Community Reintegration among Community-Dwelling Subacute Stroke Subjects: A Cross-Sectional Study. Crit Rev Phys Rehabil Med. 2024;36(2):1–11. doi: 10.1615/critrevphysrehabilmed.2023049866

[16] Wade DT, Hewer RL. Functional abilities after stroke: measurement, natural history and prognosis. J Neurol Neurosurg Psychiatry. 1987;50(2):177–82. doi: 10.1136/jnnp.50.2.177 3572432 PMC1031489

[17] Wood-Dauphinee SL, Opzoomer MA, Williams JI, Marchand B, Spitzer WO. Assessment of global function: The Reintegration to Normal Living Index. Arch Phys Med Rehabil. 1988;69(8):583–90. 3408328

[18] Bourget N, Deblock-Bellamy A, Blanchette AK, Batcho CS. Use and psychometric properties of the Reintegration to Normal Living Index in rehabilitation: A systematic review. Ann Phys Rehabil Med. 2018;61(4):262–9. doi: 10.1016/j.rehab.2017.12.004 29317299

[19] van Brakel WH, Anderson AM, Mutatkar RK, Bakirtzief Z, Nicholls PG, Raju MS, et al. The Participation Scale: measuring a key concept in public health. Disabil Rehabil. 2006;28(4):193–203. doi: 10.1080/09638280500192785 16467054

[20] Powell LE, Myers AM. The Activities-specific Balance Confidence (ABC) Scale. J Gerontol A Biol Sci Med Sci. 1995;50A(1):M28-34. doi: 10.1093/gerona/50a.1.m28 7814786

[21] Botner EM, Miller WC, Eng JJ. Measurement properties of the Activities-specific Balance Confidence Scale among individuals with stroke. Disabil Rehabil. 2005;27(4):156–63. doi: 10.1080/09638280400008982 15824045

[22] Yardley L, Beyer N, Hauer K, Kempen G, Piot-Ziegler C, Todd C. Development and initial validation of the Falls Efficacy Scale-International (FES-I). Age Ageing. 2005;34(6):614–9. doi: 10.1093/ageing/afi196 16267188

[23] Delbaere K, Close JCT, Mikolaizak AS, Sachdev PS, Brodaty H, Lord SR. The Falls Efficacy Scale International (FES-I). A comprehensive longitudinal validation study. Age Ageing. 2010;39(2):210–6. doi: 10.1093/ageing/afp225 20061508

[24] Duncan PW, Lai SM, Bode RK, Perera S, DeRosa J. Stroke Impact Scale-16: A brief assessment of physical function. Neurology. 2003;60(2):291–6. doi: 10.1212/01.wnl.0000041493.65665.d6 12552047

[25] Wang I, Wang Y-C, Wu T-Y, Chou C-Y, Hsieh C-L. Rasch Analysis of the Stroke Impact Scale-16. Am J Occup Ther. 2022;76(6):7606205130. doi: 10.5014/ajot.2022.049335 36548001

[26] Chou C-Y, Ou Y-C, Chiang T-R. Psychometric comparisons of four disease-specific health-related quality of life measures for stroke survivors. Clin Rehabil. 2015;29(8):816–29. doi: 10.1177/0269215514555137 25352615

[27] Williams LS, Weinberger M, Harris LE, Clark DO, Biller J. Development of a stroke-specific quality of life scale. Stroke. 1999;30(7):1362–9. doi: 10.1161/01.str.30.7.1362 10390308

[28] Thornton M, Travis SS. Analysis of the reliability of the modified caregiver strain index. J Gerontol B Psychol Sci Soc Sci. 2003;58(2):S127-32. doi: 10.1093/geronb/58.2.s127 12646602

[29] Zimet GD, Powell SS, Farley GK, Werkman S, Berkoff KA. Psychometric characteristics of the Multidimensional Scale of Perceived Social Support. J Pers Assess. 1990;55(3–4):610–7. doi: 10.1080/00223891.1990.9674095 2280326

[30] Nayak A, Bhave AC, Misri Z, Unnikrishnan B, Mahmood A, Joshua AM, et al. Facilitators and barriers of community reintegration among individuals with stroke: a scoping review. European Journal of Physiotherapy. 2023;25(5):291–304. doi: 10.1080/21679169.2022.2156599

[31] Alawieh A, Zhao J, Feng W. Factors affecting post-stroke motor recovery: Implications on neurotherapy after brain injury. Behav Brain Res. 2018;340:94–101. doi: 10.1016/j.bbr.2016.08.029 27531500 PMC5305670

[32] Baseman S, Fisher K, Ward L, Bhattacharya A. The relationship of physical function to social integration after stroke. J Neurosci Nurs. 2010;42(5):237–44. doi: 10.1097/jnn.0b013e3181ecafea 20968219

[33] Yang S, Boudier-Revéret M, Kwon S, Lee MY, Chang MC. Effect of Diabetes on Post-stroke Recovery: A Systematic Narrative Review. Front Neurol. 2021;12:747878. doi: 10.3389/fneur.2021.747878 34970205 PMC8712454

[34] Maïer B, Kubis N. Hypertension and Its Impact on Stroke Recovery: From a Vascular to a Parenchymal Overview. Neural Plast. 2019;2019:6843895. doi: 10.1155/2019/6843895 31737062 PMC6815533

[35] Hausdorff JM, Herman T, Baltadjieva R, Gurevich T, Giladi N. Balance and gait in older adults with systemic hypertension. Am J Cardiol. 2003;91(5):643–5. doi: 10.1016/s0002-9149(02)03332-5 12615286

[36] Woodman P, Riazi A, Pereira C, Jones F. Social participation post stroke: a meta-ethnographic review of the experiences and views of community-dwelling stroke survivors. Disabil Rehabil. 2014;36(24):2031–43. doi: 10.3109/09638288.2014.887796 24597937

[37] Honado AS, Atigossou OLG, Roy J-S, Daneault J-F, Batcho CS. Relationships between Self-Efficacy and Post-Stroke Activity Limitations, Locomotor Ability, Physical Activity, and Community Reintegration in Sub-Saharan Africa: A Cross-Sectional Study. Int J Environ Res Public Health. 2023;20(3):2286. doi: 10.3390/ijerph20032286 36767651 PMC9915935

[38] Ogunlana MO, Oyewole OO, Fafolahan A, Govender P. Exploring community reintegration among Nigerian stroke survivors. S Afr J Physiother. 2023;79(1):1857. doi: 10.4102/sajp.v79i1.1857 37415852 PMC10319923

[39] Lillekroken D, Halvorsrud L, Bjørge H, Gandhi S, Sivakumar PT, Goyal AR. Caregivers’ experiences, challenges, and needs in caring for people with dementia in India: a scoping review. BMC Health Serv Res. 2024;24(1):1661. doi: 10.1186/s12913-024-12146-x 39734191 PMC11684304

[40] Walsh ME, Galvin R, Loughnane C, Macey C, Horgan NF. Factors associated with community reintegration in the first year after stroke: a qualitative meta-synthesis. Disabil Rehabil. 2015;37(18):1599–608. doi: 10.3109/09638288.2014.974834 25382215

[41] Wei L, Zhao X, Chen X, He Y, Liu J, Xian J, et al. Caregiver Burden and Its Associated Factors Among Family Caregivers of Hospitalized Patients with Neurocritical Disease: A Cross-Sectional Study. J Multidiscip Healthc. 2024;17:5593–603. doi: 10.2147/JMDH.S492890 39619164 PMC11608030

